# ASSERT (Acute Sacral inSufficiEncy fractuRe augmenTation): Perceptions in the Assessment and Treatment of Pubic Rami and Sacral Fragility Fractures Amongst Healthcare Professionals in Geriatric Medicine and Surgery—A Qualitative Study

**DOI:** 10.1177/21514593211026794

**Published:** 2021-07-09

**Authors:** Opinder Sahota, Paul Leighton, Maribel Cameron, Rachael Taylor, Terence Ong, Avril Drummond, Paul Hendrick, Nasir Quraishi, Khalid Salem, On Behalf of the ASSERT Research Team

**Affiliations:** 1 Department of HCOP, Nottingham University Hospitals NHS Trust, Nottingham, United Kingdom; 2 The Centre for Spinal Studies and Surgery, Nottingham University Hospitals NHS Trust, Nottingham, United Kingdom; 3 University of Nottingham, Nottingham, United Kingdom; 4 National Institute for Health Research (NIHR) Nottingham Biomedical Research Centre (BRC), Nottingham, United Kingdom; 5 Faculty of Medicine, University of Malaya, Kuala Lumpur, Malaysia

**Keywords:** pubic rami fracture, sacral fracture, older people, spinal surgeons, sacral augmentation

## Abstract

**Background:**

Pubic rami fragility fractures are common in older people and result in significant morbidity and increased mortality. Co-existing fractures of the sacrum are common, but routinely missed. The aim of the study was to explore the perceptions in the assessment and treatment of pubic rami and sacral fragility fractures amongst healthcare professionals.

**Methods:**

We interviewed 14 participants about their experience in the assessment and treatment of patients presenting with pubic rami fragility fractures. Data was analyzed using an inductive thematic approach.

**Results:**

The majority of patients presenting with a pubic rami fragility fracture were managed by geriatricians. However, many of the geriatricians were not aware that these fractures have a high association with co-existing sacral fragility fractures. Furthermore, they were not aware of the limitations of standard x-ray imaging, nor of the potential benefits of surgical intervention for sacral fragility fractures. Spinal surgeons recommended that early, more specialist imaging in patients with pubic rami fragility fractures failing to mobilize, would change clinical management, if found to have a coexisting sacral fragility fracture, amenable to surgical intervention.

**Conclusions:**

The awareness, assessment and management of sacral fragility fractures in patients presenting with pubic rami fragility fractures is poor amongst healthcare professionals in geriatric medicine. Spinal surgeons in this study advocate early further imaging and surgical intervention in patients confirmed to have a concomitant sacral fragility fracture who are failing to mobilize.

## Background

Pelvic Fragility Fractures (PFF) are common in older people and occur as a result of a low-energy trauma, typically following a fall from standing height or less.^1-4^ The overall incidence is 92/100,000 per year in those aged 65 years and older^5^ rising to 450/100,000 in those 80 years and over.^6^ PFF can be classified into fractures of the anterior pelvic ring (pubic rami fragility fractures), or fractures of the posterior pelvic ring (sacral fragility fractures). Up to 60% of older patients presenting with a pubic rami fragility fracture will have a concurrent fracture of the sacrum.^7-9^ The mean hospital length of stay is longer for those with concurrent pubic rami and sacral fragility fractures,^10^ with greater dependency and higher rates of institutionalization,^11^ compared to those with an isolated pubic rami fragility fracture.

The body’s line of gravity is situated very slightly behind the femoral heads, and frontally runs through the middle of the sacrum, at a point equidistant from the 2 femoral heads.^12^ This results in 70% of load bearing through the posterior pelvis and 30% anteriorly. Thus isolated pubic rami fractures can be managed conservatively, with non-surgical treatment. However, even when seated, the load bearing on the sacrum is significant, and in the presence of a sacral fragility fracture, is extremely painful. Key-hole, minimally invasive surgery (sacroplasty) is routinely available for the treatment of sacral fragility fractures and has been shown to be safe, effective for pain relief and improves mobility when compared to conservative (non-surgical) treatment.^13-15^

The difficulty in managing sacral fragility fractures lies in their diagnosis, since they are not easily identifiable on conventional pelvic radiographs, due to generalized osteoporosis, overlying bowel shadows, obliquity of the sacrum and vascular calcifications.^16^ The number of sacral fragility fractures detected on pelvic radiographs alone ranges from 0 to 10%.^17^ Computerized tomography (CT) is the preferred imaging modality for detecting sacral fragility fractures, but is not considered as a standard investigation in patients presenting with pubic rami fragility fractures. When CT-scanning is used as standard, sacral fragility fractures rates of up to 80% are reported, depending on the definition of the fracture used.^18^ Not knowing the full extent of the fractures could be one of the reasons why some patients’ experience unexplained, prolonged pain and consequent debilitation during conservative (non-surgical) treatment. Non-surgical treatment of a painful sacral fragility fracture may lead to progressive instability, non-union, and secondary fragility fractures. Complications of immobilization are seen in 20–58% of patients^19-20^ with high mortality rates.^21-22^ Therefore, operative intervention to reduce pain, reduce immobilization, and consequent debilitation should be considered as part of routine care, in selected patients.^17^

The aim of the study was to explore perceptions in the assessment and treatment of pubic rami and sacral fragility fractures amongst health care professionals in geriatric medicine and surgery in a busy teaching hospital.

## Methods

### Qualitative Interview Study Utilizing Semi-Structured Interviews

#### Study *setting and participants*

This study was nested within the ASSERT study,^23^ a feasibility study supporting the development of a clinical and cost-effectiveness trial of keyhole surgery of sacral insufficiency fractures compared to non-surgical (conservative) treatment.

Participants were drawn from the geriatric medicine and surgery department involved in the management of pubic rami and sacral fragility fractures, in a large UK teaching hospital. Sampling was driven by convenience, although effort was made to capture the perspectives of a range of healthcare professionals in geriatric medicine and spinal surgery to capture diverse clinical and surgical perspectives.

#### Data collection

Participants were invited to take part in a 30-45 mins semi-structured interview.

Interviews were conducted to consider 3 broad topics:

- clinical presentation of the pubic rami fragility fracture
- awareness and identification of sacral fragility fractures
- treatment options for sacral fragility fractures

Specific areas of interest included prior experience of managing these fragility fractures and views regarding treatment and treatment success in the older population (see Online Appendix 1 interview guide). All interviews were undertaken face-to-face, by a research nurse (RGN, female), experienced in qualitative techniques, at a time and location convenient to the participant, in the hospital. No repeat interviews were conducted.

Interviews were digitally recorded using an audio-recorder and transcribed in full. No field notes were taken at the time of the interviews and transcripts were not returned to the participants for further comments. Transcripts were anonymized and data stored on a password protected, networked drive. Interview data was handled using the N’Vivo (version 11)^24^ software package.

### Data Analysis

A framework approach to thematic analysis was adopted^25-26^ and analytic matrices, informed by the research feasibility aims constructed (see Online Appendix 2 analytic table). Separate matrices were constructed for surgical and medical healthcare participants in order to reflect (and illuminate) their different perspectives.

Interview data were mapped to these matrices. Mapping of data was checked and validated by the broader author group to ensure appropriate interpretation of the insights offered. Matrices, and elements of the matrices were summarized and comparisons were made between health care professionals in geriatric medicine and spine surgery.

## Results

### Participants

Thirty-two healthcare professionals in geriatric medicine, spinal surgery and orthopaedics were invited to participate, 14 of whom agreed to participate. The reasons for non-participation were documented where possible; the main reasons given were lack of time and conflict of schedule.

The characteristics of recruited participants are shown in Table 1.

**Table 1.** Characteristics of Interviewed Participants.

Participant ID	Department	Speciality
001	Spinal	Spinal Surgeon
002	Geriatric Medicine	Geriatrician
003	Geriatric Medicine	Geriatrician
004	Geriatric Medicine	Geriatrician
005	Spinal	Spinal Physiotherapy
006	Geriatric Medicine	Geriatrician
007	Spinal	Advanced Nurse Practitioner
008	Geriatric Medicine	Advanced Nurse Practitioner
009	Spinal	Spinal Surgeon
010	Spinal	Spinal Surgeon
011	Spinal	Spinal Surgeon
012	Spinal	Spinal Surgeon
013	Geriatric Medicine	Advanced Nurse Practitioner
014	Geriatric Medicine	Geriatrician

### Clinical Presentation of the Pubic Rami Fragility Fracture

The majority of patients admitted to hospital with a pubic rami fracture were admitted under a geriatrician in a seemingly straightforward, uncomplicated fashion (003-Geriatric Medicine (GM); 009-Spinal (S)). Concerns for other fractures (e.g. hip) were a more likely initial concern and no focus upon sacral fragility fractures was evident in the responses offered by those from geriatric medicine:

“They come up and they’ve had a hip fracture ruled out or they’ve not had any x-rays yet and then it’s when they’re up and mobilizing and they’re still in a lot of pain and they describe the pain to you then sometimes further imaging is taken, so pelvic x-rays and that’s when they often identify them having a pubic rami fracture” (013 – GM)

“If someone who came in with a fall and complaining of problem with especially with groin/hip pain, then we have to find that suspicion to start with. So a lot of times we concentrate on hip fracture because that’s a problem with that. So I’ll think about that, I will arrange an x-ray’ (006-GM)

### Awareness of Sacral Fragility Fractures

Participants from geriatric medicine acknowledged their limited awareness of sacral fragility fractures; “*I knew they can occur…I wasn’t aware that the incidence was high”* (004-GM). Rather they described pursuing a conservative approach to managing pubic rami fractures with little concern that other injuries might be present. Consultation with a surgeon would more likely be a “*courtesy*”, rather than undertaken in the expectation that action is required or likely to result:

“If the patient just had a single pubic rami fracture in the first place then you would let them mobilize. We usually talk to the orthopaedic surgeons first, but we just kind of do that as a courtesy and they always say ‘mobilize unless pain allows, unless they’ve got lots of fractures going on’ and we then mobilize them, we see how they are with their mobility, how comfortable they are, how much analgesia they need” (008-GM)

In contrast participants from the spinal department recognized the common coincidence of pubic rami and sacral fragility fractures: “*most patients with acute rami fractures will have some element of sacral fracture because it’s a ring”* (001-S); *“Patients with a pubic rami fragility fracture, they definitely have a higher incidence of the sacral fragility fracture”* (009-S). Awareness of this coincidence of injuries means that those from the spinal department are also more likely to be aware of those symptoms which typify a sacral fragility fracture: “*clinical suspicion of a sacral fragility fracture, which is more of a lower back pain”* (010-S).

### Identifying Sacral Fragility Fractures

The conservative (non-surgical) approach adopted by those in geriatric medicine was typified by a concern for patient mobility and the pain that they experience, additional consultation only being considered where improvements in mobility and pain are not manifest. The orthopaedic department might be an avenue for such advice, and here participants from geriatric medicine described that a conservative approach would generally be reinforced.

Additional diagnostic imaging might be sought where improvements have not been seen, although this in part might be to ensure that a hip fracture has not been missed:

“If they do appear to be in significant pain, we might review the x-rays, we might do a CT scan, or they need an MRI for a hip fracture that’s not revealed itself or something down the line” (003-GM)

“ If after 72 hours they’re still really struggling with the mobility and they’re struggling with pain we would then consider further imaging, so we’d think about doing a CT or an MRI” (008-GM)

In contrast, consultation with the spinal team is more likely to recommend immediate imaging in order to exclude a coexisting sacral fragility fracture:

“So pain that, maybe it’s hip pain, pain that goes to the backside, we’ll say that there is something more than just a pubic pain that would be more of a kind like, more in pain. And usually those fractures, you cannot see quite clearly from an x-ray, so the next step will be an MRI” (001-S)

“If I press on an area and the patient winces in pain then that tells me something is really wrong there. So then we do an MRI scan for somebody who is having difficulty mobilizing or reporting very significant pain” (012-S)

“For diagnosing sacral fractures it’s usually MRI because most often…the first presentations won’t [show] and they are often not seen on CT scan” (009-S)

### Treatment of Sacral Fragility Fractures

Those in geriatric medicine demonstrated a systematic approach to managing pubic rami fragility fractures primarily focused upon pain management and informed by NICE and WHO guidelines:

“If x-ray shows pubic ramus fracture then the first thing is pain management. So I tend to use the WHO Analgesics Ladder, trying to control the pain to allow them to walk as soon as possible. And hopefully they will be able to mobilize.” (006-GM)

“So analgesia and that obviously depends on the patients, intravenous paracetamol’s quite commonly used and we try to avoid opiates in the older frail patients. But often if they’re assumed to be a fractured neck of femur, if they have significant pain, so they get opiates. If it’s an isolated injury, it’s simple analgesia, try with mobilization and if they can mobilize adequately and safely then discharge home. And if they fail to mobilize they’ll be admitted to our rehabilitation ward, for ongoing analgesia and mobilization, physiotherapy” (003-GM)

As with their awareness of sacral fragility fractures, the awareness of the sacroplasty procedure amongst those in geriatric medicine was limited: “*If it shows a sacral fracture, I’ll still be managing their pain conservatively with pain killers. I’ll try and increase that and control the pain”* (006-GM); *“Well, up till recently I thought that there was not much of apart from analgesia”* (004-GM).

Where a sacral fragility fracture is confirmed, and where patients are failing to mobilize, those in the spinal department recognized the potential that the sacroplasty offers: “*outcomes are generally good*”, “*much better than conservative management*” (011-S). This procedure is perceived to offer better pain management outcomes:

“Patients who have sacroplasty are literally more pain free compared to those with non-operative treatment somehow—I don’t know why—but again I’m not sure about the statistical analysis and how does that mean, but they’re slightly better than the non-operative treatment” (009-S)

“Because sacroplasty is essentially kind of, in a very literal term, glues the fracture together, so the pain generation is significantly reduced. I mean to compare sacroplasty and conservative treatment, I would feel sacroplasty patients do have a much more rapid improvement in their pain scores” (010-S)

More than improvements in pain, spinal specialists associated sacroplasty with more general health benefits—or rather the absence of physical decline associated with extended immobility: “*it’s other things…conservative can include pneumonia,* [and] *other sources of infection”* (011-S); “*there is no point keeping them long in the bed because we know they just end up with more and more problems*” (009-S).

## Discussion and Implications

The study explored perceptions in the assessment and treatment of pubic rami and sacral fragility fractures amongst health care professionals in geriatric medicine and surgery. We found, from the geriatricians interviewed that they felt that the majority of patients presenting to hospital with a pubic rami fragility fracture were admitted under and cared for by a geriatrician. However, there was poor awareness of the high association of co-existing sacral insufficiency fractures in this group and poor recognition of the limitations of standard x-ray imaging, (sacrum cannot be seen). This is not unsurprising, since the recognition and management of sacral fragility fractures lies in their diagnosis. These fractures are not easily identifiable on conventional pelvic radiograph^16^ and even when seen on CT, retrospectively, the number of sacral fragility fractures detected on pelvic radiographs ranges from 0 to 10%.^17^ Computerized tomography (CT) is the preferred imaging modality for detecting sacral fragility fractures, but is not considered as a standard investigation in patients presenting with pubic rami fragility fractures.

By contrast, health care professionals interviewed in spine surgery were well aware of the co-exiting, high association of sacral fragility factures in patients presenting with pubic rami fragility fractures. They recommend early MR imaging and commented on how this would change clinical management. CT is recommended as the investigation of choice,^26^ although currently there are no national UK guidelines for the management of these fractures. The spine surgeons we interviewed preferred MR imaging, since this would highlight the presence of sacral oedema, at the site of an osseous trabecular fracture, irrespective of cortical breach-this feature is highly indicative of acute injury. However, the limitations to timely MR in the UK are well recognized. Yet, arguably, given the high prevalence of combined pubic rami and sacral insufficiency fractures, and the potential for surgical intervention, these patients perhaps should be admitted under the care of an orthopaedic / spinal surgeon and not a geriatrician?

Spinal surgeons interviewed advocated early sacroplasty for patients confirmed with a sacral fragility fracture and unable to mobilize. Key-hole, minimally invasive surgery (sacroplasty) has been shown to be safe, effective for pain relief and improves mobility when compared to conservative (non-surgical) treatment.^13-15^ A meta-analysis by Chandra et al,^13^ which included 19 trials, demonstrated statistically significant differences in the visual analogue scale (VAS) pain level at pre-procedure, 24-48 hours, 6 months, and 12 months follow up, with cumulative pain scores of 8.32 ± 0.01, 3.55 ± 0.01, 1.48 ± 0.01, and 0.923 ± 0.01, respectively. The pooled major complication rate from the intervention was small at 0.3%. Similarly in a systematic review, Mahmood et al,^14^ reported the mean reduction in pain score from pre-procedure to latest follow-up post-procedure VAS to be 5.8+1.3, while the risk of cement extravasation (the most commonly reported complication), clinically insignificant in the majority of studies included. Two studies reported S1 radicular pain after the procedure while only one study reported a patient with persistent pain requiring re-operation. Most of the healthcare professionals in geriatric medicine interviewed in our study were unaware of this procedure.

Improving the care of older people is a high priority for the NHS, and in particular those with frailty.^27^ More recently, the Specialized Clinical Frailty Network (a funded collaborative program delivered by NHS England in partnership with NHS Elect), which delivers a program of support framed around the early identification of frailty and the development of frailty focused care pathways has identified Neurosurgery, Spinal Surgery and Adult Critical Care services as part of the Wave II national program.^28^ Improving the care of older people with fragility fractures has been highlighted by a number of specialist societies across the UK.^29^ However, we still lack clear, national guidance on the management of PFF in older people, as national policy documents relating to pelvic fracture remain predominately focused on the management of high energy traumatic pelvic fractures, which although valuable, does not inform the management low energy PFF.^30^

## Strengths and Limitations

A important limitation of the study was that we did not interview orthopedic surgeons. In many units across the country, the first point of referral from a medical team is to the orthopedic team and spinal surgeons are often part of a tertiary hospital service. Spinal surgeons manage the spine, including the sacrum and thus the views and expertise in managing sacral fractures may differ between orthopedic and spinal surgeons. We invited 5 orthopedic surgeons to take part, but they did not respond to the invite. Further research is needed in this area.

However, a major strength of this study is that we included an equal mix of health care professional in geriatric medicine and spinal surgery. In addition, we interviewed participants in geriatric medicine, who were not ortho-geriatricians, thus more reflective of the ‘jobbing’ general geriatrician and thus more generalizable to the UK setting.

## Conclusion

The awareness, assessment and management of sacral fragility fractures inpatients presenting with pubic rami fragility fractures are poor amongst health care professionals in geriatric medicine. Spinal surgeons interviewed advocated early further imaging in patients confirmed to have a public rami fragility fracture with ongoing severe patient, refractory to standard analgesia, to exclude the presence of a concomitant sacral fragility fracture. In those patients failing to mobilize, with a confirmed sacral fragility fracture, spinal surgeons interviewed advocated surgical (sacroplasty) intervention.

## Supplemental Material

Supplemental Material, sj-pdf-1-gos-10.1177_21514593211026794 - ASSERT (Acute Sacral inSufficiEncy fractuRe augmenTation): Perceptions in the Assessment and Treatment of Pubic Rami and Sacral Fragility Fractures Amongst Healthcare Professionals in Geriatric Medicine and Surgery—A Qualitative Study Click here for additional data file. Supplemental Material, sj-pdf-1-gos-10.1177_21514593211026794 for ASSERT (Acute Sacral inSufficiEncy fractuRe augmenTation): Perceptions in the Assessment and Treatment of Pubic Rami and Sacral Fragility Fractures Amongst Healthcare Professionals in Geriatric Medicine and Surgery—A Qualitative Study by Opinder Sahota, Paul Leighton, Maribel Cameron, Rachael Taylor, Terence Ong, Avril Drummond, Paul Hendrick, Nasir Quraishi, Khalid Salem and On Behalf of the ASSERT Research Team in Geriatric Orthopaedic Surgery & Rehabilitation

Supplemental Material, sj-pdf-2-gos-10.1177_21514593211026794 - ASSERT (Acute Sacral inSufficiEncy fractuRe augmenTation): Perceptions in the Assessment and Treatment of Pubic Rami and Sacral Fragility Fractures Amongst Healthcare Professionals in Geriatric Medicine and Surgery—A Qualitative Study Click here for additional data file. Supplemental Material, sj-pdf-2-gos-10.1177_21514593211026794 for ASSERT (Acute Sacral inSufficiEncy fractuRe augmenTation): Perceptions in the Assessment and Treatment of Pubic Rami and Sacral Fragility Fractures Amongst Healthcare Professionals in Geriatric Medicine and Surgery—A Qualitative Study by Opinder Sahota, Paul Leighton, Maribel Cameron, Rachael Taylor, Terence Ong, Avril Drummond, Paul Hendrick, Nasir Quraishi, Khalid Salem and On Behalf of the ASSERT Research Team in Geriatric Orthopaedic Surgery & Rehabilitation
