# Supplementary material for: ASSERT (Acute Sacral inSufficiEncy fractuRe augmenTation): Perceptions in the Assessment and Treatment of Pubic Rami and Sacral Fragility Fractures Amongst Healthcare Professionals in Geriatric Medicine and Surgery—A Qualitative Study
Source: Geriatr Orthop Surg Rehabil. 2021 Jul 9;12:21514593211026794. doi: 10.1177/21514593211026794 (PMC8273401; doi:10.1177/21514593211026794)
Supplement: Supplemental Material, sj-pdf-1-gos-10.1177_21514593211026794 - ASSERT (Acute Sacral inSufficiEncy fractuRe augmenTation): Perceptions in the Assessment and Treatment of Pubic Rami and Sacral Fragility Fractures Amongst Healthcare Professionals in Geriatric Medicine and Surgery—A Qualitative Study [file sj-pdf-1-gos-10.1177_21514593211026794.pdf]

ASSERT (Acute Sacral inSufficiEncy fractuRe augmenTation): Perceptions in the assessment and treatment of pubic rami and sacral fragility fractures amongst healthcare professionals in geriatric medicine and surgery-a qualitative study.

Appendix 1

<u>Analytic Table for Surgeons</u>

Include only interview data collected from surgeons in this table.

INT	Comment on the clinical population.	Timing of procedure.	Description / assessment of procedure.	Difficulties of the Treatment.	Assessment of clinical success/failure. [Surgical]	Assessment of clinical success/failure. [Conservative]	Other interesting comments.
CL0x							
CL0x							
CL0x							
CL0x							
CL0x							
CL0x							
CL0x							

Analytic Table for Clinicians

Include only interview data collected from other clinicians in this table.

INT	Awareness of fracture.	Perception of the clinical population.	Treatment options and assessment.	Other interesting comments.
CL0x				
CL0x				
CL0x				
CL0x				
CL0x				
CL0x				
CL0x				
