# Supplementary material for: ASSERT (Acute Sacral inSufficiEncy fractuRe augmenTation): Perceptions in the Assessment and Treatment of Pubic Rami and Sacral Fragility Fractures Amongst Healthcare Professionals in Geriatric Medicine and Surgery—A Qualitative Study
Source: Geriatr Orthop Surg Rehabil. 2021 Jul 9;12:21514593211026794. doi: 10.1177/21514593211026794 (PMC8273401; doi:10.1177/21514593211026794)
Supplement: Supplemental Material, sj-pdf-2-gos-10.1177_21514593211026794 - ASSERT (Acute Sacral inSufficiEncy fractuRe augmenTation): Perceptions in the Assessment and Treatment of Pubic Rami and Sacral Fragility Fractures Amongst Healthcare Professionals in Geriatric Medicine and Surgery—A Qualitative Study [file sj-pdf-2-gos-10.1177_21514593211026794.pdf]

ASSERT – interview topic guide (health care professional) ver. 1.0

INTERVIEW at Wk1

A loosely structured interview intended to enable health care professionals to comment upon ASSERT and make recommendations for future research study design.

- ASSERT question.

  It would be nice to hear your assessment of the ASSERT research question.
  prompts – Why did you agree to take part in ASSERT? How important do you think this topic is? What is the clinical potential/ benefit for a full clinical trial?

  Are you happy that we are targeting the right patient group?
  prompts – Were you happy to randomise your patients? Do you think our inclusion/exclusion criteria are appropriate? Did you have any misgivings about this – generally or with specific patients?

- ASSERT processes.

  It would be nice to hear your assessment of the ASSERT research processes.
  prompts – how well do you think they have worked here? Have you struggled with any at any point? What impact upon your clinical processes have they had? What might be done about this? Do you think a larger study is feasible using these processes?

  Do you feel that we are collecting the right data and using the right instruments for data collection?
  prompts – are there any other clinical or PROM measures that you feel would be beneficial in a future study? Do you think that we have the timetable of data collection right? Was early (on ward) collection of data feasible in your setting?

  Are there any mechanism/ resources that would make ASSERT easier to deliver in clinical practice?
  prompts – what changes could we make to make it easier? Would dedicated staff help?

- Close.

  Is there anything else that you would like to raise or to make us aware of about the ASSERT study in your clinical setting?

  Summarise / seek clarification on any key point or any area of uncertainty.

  Offer opportunity for interviewee to ask questions.
